# Characterization and Parametric Study on Mechanical Properties Enhancement in Biodegradable Chitosan-Reinforced Starch-Based Bioplastic Film

**DOI:** 10.3390/polym14020278

**Published:** 2022-01-11

**Authors:** Shiou Xuan Tan, Hwai Chyuan Ong, Andri Andriyana, Steven Lim, Yean Ling Pang, Fitranto Kusumo, Gek Cheng Ngoh

**Affiliations:** 1 Department of Mechanical Engineering, Faculty of Engineering, Universiti Malaya, Kuala Lumpur 50603, Malaysia; kva190014@siswa.um.edu.my (S.X.T.); andri.andriyana@um.edu.my (A.A.); 2 Future Technology Research Center, National Yunlin University of Science and Technology, Douliou 64002, Taiwan; 3 Department of Chemical Engineering, Lee Kong Chian Faculty of Engineering and Science, Universiti Tunku Abdul Rahman, Kajang 43000, Malaysia; pangyl@utar.edu.my; 4 Centre of Photonics and Advanced Materials Research, Universiti Tunku Abdul Rahman, Kajang 43000, Malaysia; 5 Centre for Green Technology, School of Civil and Environmental Engineering, University of Technology, Sydney, NSW 2007, Australia; Fitranto.Kusumo@student.uts.edu.au; 6 Department of Chemical Engineering, Faculty of Engineering, Universiti Malaya, Kuala Lumpur 50603, Malaysia

**Keywords:** starch-based bioplastic, chitosan, co-polymer, reinforcement, biodegradation

## Abstract

Bioplastic has been perceived as a promising candidate to replace petroleum-based plastics due to its environment-friendly and biodegradable characteristics. This study presents the chitosan reinforced starch-based bioplastic film prepared by the solution casting and evaporation method. The effects of processing parameters, i.e., starch concentration, glycerol loading, process temperature and chitosan loading on mechanical properties were examined. Optimum tensile strength of 5.19 MPa and elongation at break of 44.6% were obtained under the combined reaction conditions of 5 wt.% starch concentration, 40 wt.% glycerol loading, 20 wt.% chitosan loading and at a process temperature of 70 °C. From the artificial neural network (ANN) modeling, the coefficient of determination (R^2^) for tensile strength and elongation at break were found to be 0.9955 and 0.9859, respectively, which proved the model had good fit with the experimental data. Interaction and miscibility between starch and chitosan were proven through the peaks shifting to a lower wavenumber in FTIR and a reduction of crystallinity in XRD. TGA results suggested the chitosan-reinforced starch-based bioplastic possessed reasonable thermal stability under 290 °C. Enhancement in water resistance of chitosan-incorporated starch-based bioplastic film was evidenced with a water uptake of 251% as compared to a 302% registered by the pure starch-based bioplastic film. In addition, the fact that the chitosan-reinforced starch-based bioplastic film degraded to 52.1% of its initial weight after 28 days suggests it is a more sustainable alternative than the petroleum-based plastics.

## 1. Introduction

Petroleum-based plastics have constituted major parts of our daily life, ranging from bottled drinks, household appliances and toys to food packaging. The reasons for plastics having such popular usage are due to their high strength, low cost and light-weight as well as their thermal and chemical insulating properties [1,2]. However, petroleum-based plastics are resistant to chemical, solar and microbial degradation [1,3]. This leads to a build-up of plastic waste in the environment along with increasing utilization of plastic products [4,5]. The shortcomings can be addressed by the emergence of bioplastics.

Bioplastics’ renewable nature is associated with constituents that are derived from plants such as starch, cellulose and lignin or from animal-derived casein, protein and lipid [6]. Bioplastics have an edge over petroleum-based plastics due to their biodegradability, instead of the combustion of plastic wastes adopted by the latter [7,8]. Among the diverse feedstocks for synthesizing of bioplastic, starch is preferred, as it can be easily degraded into environment-friendly compounds [9]. In addition, its affordability, abundance and availability in various starch-producing plants such as sago, cassava, corn and potato have also added to its superior advantages. However, pure starch-based bioplastic film is hydrophilic and highly sensitive to water, which would negatively affect its mechanical and barrier properties, and thereby greatly limit its utilization [10,11,12].

In starch-based bioplastic film fabrication, plasticizer can be used to overcome film brittleness caused by the high intermolecular forces [13,14]. The commonly used plasticizers for starch-based bioplastic film are water, sorbitol and glycerol [15]. Nevertheless, it is not recommended for water to be used directly as plasticizer in view of the high volatility of its molecules, which would lead to brittleness in film [16,17]. Glycerol, conversely, possesses hydroxyl groups that are responsible for inter- and intra-molecular interactions (hydrogen bonds) in polymeric chains. Thus, it is regarded as the best plasticizer for water-soluble polymers in providing a more flexible structure for bioplastic films [5,13,18].

The properties of pure starch-based bioplastic film could also be enhanced by blending it with other polymers such as chitosan with its hydrophobic nature [19,20]. Chitosan is obtained from the de-acetylation of chitin, which could be abundantly found in natural sources such as shells and heads of crabs, lobsters or shrimps [21,22]. The non-toxic and low-cost nature of chitosan has made it a suitable choice as a co-polymer in bioplastic film fabrication for improving mechanical properties, while reducing the hydrophilic characteristic of starch-based bioplastic film [19]. In addition, the bioplastic film produced from blending of starch with chitosan would be very attractive as a food-packaging application due to the intrinsic antimicrobial property of chitosan. Starch-based bioplastic films have been synthesized by many researchers from various sources, such as potato [23], corn [24,25], mango seed [26], jackfruit seed [27], sago [21], avocado seed [28] and cassava peel [29]. Despite bioplastic fabrication being conducted by many researchers, the parametric studies on plasticizer and filler/co-polymer loadings using solution and casting techniques are rarely being conducted. Possibly, this has been due to the restrictions of smaller scale (laboratory scale), which was less developed in its present status. Moreover, the results obtained had not been proven for industrial scale compared to other matured techniques such as extrusion, blow molding and compression molding. The effects of starch concentration and process temperature on the mechanical properties of bioplastic film are also seldom being investigated and their relationships are still unclear. Hence, this paper aims to provide more insights into the effects of operating parameters, including starch concentration, glycerol loading, process temperature and chitosan loading on the mechanical properties of the synthesized bioplastic film, based on the solution casting and evaporation technique. The present work employs corn starch as matrix, chitosan as co-polymer and glycerol as plasticizer in the fabrication of a sustainable bioplastic film. The results of this parametric study on the mechanical properties of the starch-based bioplastic film will develop a fundamental framework for more in-depth studies in the future. Artificial neural network (ANN) was adopted in this study in order to compare the experimental and predicted tensile strength and elongation at break. In addition, pure starch-based and chitosan-reinforced starch-based bioplastic films were compared in terms of their water uptake capability and actual biodegradability in soil to address the future of applicability of bioplastics.

## 2. Materials and Methods

### 2.1. Materials

Corn starch powder was procured from Thye Huat Chan Sdn. Bhd., Malaysia. Glycerol (99.5% purity) was procured from Friendemann Schmidt. Acetic acid (glacial, 100% purity) and chitosan (from shrimp shells, medium molecular weight) were purchased from Merck and Sigma-Aldrich, respectively. Laboratory-formulated distilled water was used throughout the experiments. All the chemicals were used as received, without further purification.

### 2.2. Preparation of Corn Starch Bioplastic Film

**Figure 1:**
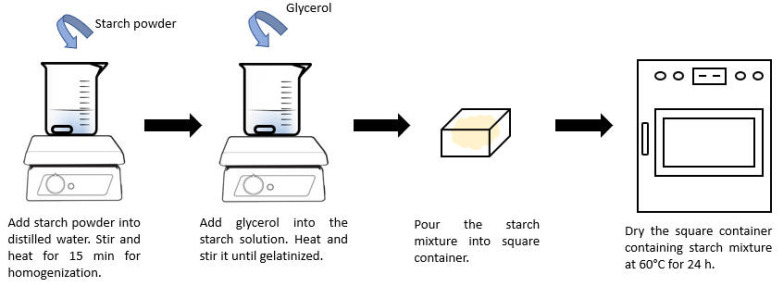
Experimental setup for bioplastic film fabrication.

The solution casting and evaporation method was adopted for bioplastic film preparation in this study. The formulation was modified from the work of Salehudin et al. [30]. A schematic diagram of the experimental setup is shown in Figure 1. First, a varying amount of corn starch (2.5 wt.%–10 wt.% of distilled water), which corresponded to starch: glycerol ratio (*w*/*w*) of 10:8–40:8 was added into a 250 mL beaker containing 100 mL distilled water in a form of suspension to avoid agglomeration. The starch solution was homogenized at 250 rpm and heated at 60 °C for 15 min. Glycerol of varying weight percentages (40 wt.%–80 wt.% based on weight of starch only) was added into the starch solution and subjected to different process temperatures (65 °C–90 °C) until it was gelatinized. For the addition of varying chitosan loading (0 wt.%–20 wt.% of starch), chitosan was dispersed in aqueous solution of 1 *v*/*v*% acetic acid. Upon completion of the reaction, 15 g of starch mixture or starch-chitosan mixture was poured into a square container with dimension of 10 cm × 10 cm. The mixture was then dried in a heating oven at 60 °C for 24 h. The film was peeled off and stored in a desiccator with a sealed plastic bag prior to thickness measurement and further testing. The final thickness of each film was measured and was in the range of 0.11 ± 0.02 mm. The effects of fabrication parameters, including starch concentration, glycerol loading, process temperature and chitosan loading towards the mechanical properties of corn starch bioplastic film were investigated using the one factor at a time method. Each experiment was conducted in triplicates to ensure the data reproducibility. The reported values were the average of the individual runs, with errors within an acceptable range of 5%. The pure starch-based and chitosan-reinforced starch-based bioplastic films with the optimum tensile strength were then subjected to water uptake and biodegradation tests to further characterize the bioplastic properties.

### 2.3. ANN Modeling

The ANN model was developed using MATLAB Version 7.10 R2010a software (The MathWorks Inc., Natick, MA, USA). In this study, the hyperbolic tangent sigmoid transfer function (tansig) was used for the input layer to the hidden layer and the linear transfer function (purelin) was used from the hidden layer to the output layer. Once the mean squared error (MSE) reached the minimum value and the average correlation coefficient (R) was close or equal to 1, the training of ANN model was terminated.

### 2.4. Data Verification

The performance of the ANN model was measured statistically based on the coefficient of determination (R^2^) according to Equation (1) [31]. Generally, a higher R^2^ value indicated higher accuracy for the developed model.

(1) $$R^{2}=1-\overset{n}{\underset{i=1}{\sum }}\left(\frac{{\left(y_{bi}-y_{pi}\right)}^{2}}{{\left(y_{m}-y_{pi}\right)}^{2}}\right)$$

where *n* is the number of experimental data, while *y_bi_* is the experimental tensile strength/elongation at break, *y_pi_* is the predicted tensile strength/elongation at break and *y_m_* is the average value of the tensile strength/elongation at break obtained from the experiments.

### 2.5. Characterization of Bioplastic Film

#### 2.5.1. Mechanical Properties

The mechanical properties of bioplastic film such as tensile strength and elongation at break were determined using Universal Testing Machine (Autograph AG-X, Shimadzu, Japan) interfaced with computer operating Trapezium software. Measurements were performed with load cell of 500 N, crosshead speed of 5 mm/min and grip separation of 30 mm. Three readings were taken from three random places of each film, measuring 7 cm × 1 cm. The average values from three measurements were reported.

#### 2.5.2. Fourier-Transform Infrared (FTIR) Analysis

FTIR spectra of pure starch-based and chitosan-reinforced starch-based bioplastic films were recorded using FTIR spectrometer operating in attenuated total reflectance (ATR) mode by positioning the measuring probe directly on the film surface (Perkin Elmer, model Spectrum 400) to compare the functional groups and chemical bonds between the films. FTIR spectra of corn starch and chitosan powders were also acquired by placing the samples on the test area. All the samples were scanned at a frequency range of 4000–450 cm^−1^ with 32 scans at a resolution of 4 cm^−1^.

#### 2.5.3. X-ray Diffraction (XRD)

XRD patterns were recorded using an X-ray diffractometer (Rigaku, model MiniFlex 300/600) equipped with CuKα radiation, operating at 40 kV and 14 mA. All the samples were measured at room temperature over the angular range 3–90° (2θ) at a step size of 0.02° in continuous mode.

#### 2.5.4. Thermal Analysis

Thermal behaviors of pure starch-based and chitosan-reinforced starch-based bioplastic films were characterized by thermal gravimetric analysis (TGA). This thermal decomposition analysis was performed by using a thermogravimetric analyzer (Perkin Elmer, model TGA Pyris 1). A bioplastic film of around 5 mg was placed in a platinum pan and heated from ambient temperature to a final temperature of 900 °C at a heating rate of 20 °C/min with nitrogen flow rate of 50 mL/min [19,32].

#### 2.5.5. Water Uptake Test

The water uptake test was modified from the study of Maulida and Tarigan [29] and Wu [33]. Bioplastic film was cut into a uniform size of 2 cm × 2 cm with its mass being weighed. The film was then immersed in a container filled with distilled water for 90 min. During the period, the film was removed from the water at 10-min intervals. Immediately after it was blotted dried with tissue paper, the film was weighed before returning to the water. The water uptake by the film was calculated according to Equation (2) [4]. The average values from three measurements were reported.

(2) $$\mathrm{Water}\;\mathrm{uptake}=\frac{W-W_{o}}{W_{o}}\times 100\%$$

where $\;W_{o}$ is the weight of dry sample (g), W is the weight of sample after immersion in distilled water (g)

#### 2.5.6. Biodegradation Test

Bioplastic samples with the dimension 2 cm × 2 cm were buried in compost soil purchased from a local plant nursery at ±3 cm depth and left for 28 days with weekly sampling being performed. The buried samples were cleaned of soil and weighed. The biodegradation test was conducted at room temperature with relative humidity of 40–50%. The weight loss of the bioplastics was calculated using Equation (3) [9]. Three replicates of each sample were subjected to the biodegradation test and the average values were reported.

(3) $$\mathrm{Weight}\;\mathrm{loss}=\frac{W_{o}-W_{f}}{W_{o}}\times 100\%$$

where $\;W_{o}$ is the initial bioplastic weight (g), $W_{f}$ is the final bioplastic weight (g).

### 2.6. Statistical Analysis

Statistical analysis was performed on OriginPro 2018 software (OriginLab, USA). One-way analysis of variance (ANOVA) with Tukey test was performed to compare the mean values at the significance level of 5% (*p* < 0.05).

## 3. Results and Discussion

### 3.1. Mechanical Properties

#### 3.1.1. Effect of Starch Concentration

**Figure 2:**
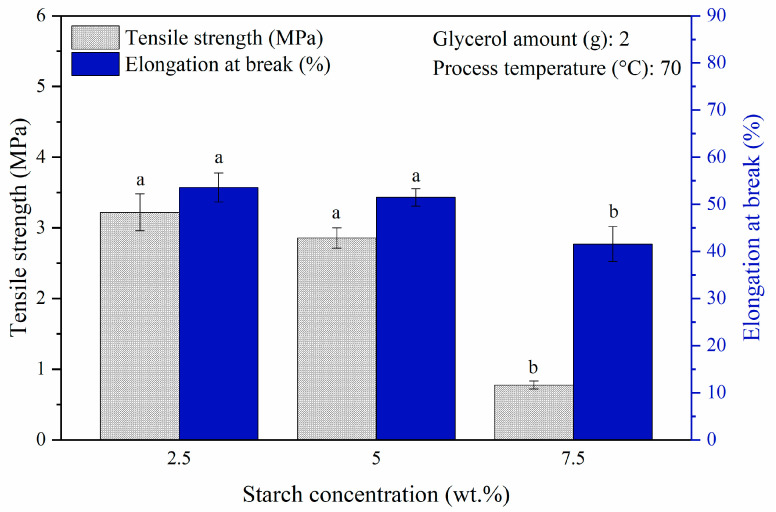
Tensile strength and elongation at break of pure starch-based bioplastic films at different starch concentrations. Different letters indicate the values are significantly different (*p* < 0.05).

The effects of starch concentration on the mechanical properties that were evaluated by fixing a glycerol amount of 2 g and a process temperature of 70 °C without incorporation of chitosan are presented in Figure 2. The tensile strength of bioplastic film decreased significantly from 3.22 MPa to 0.78 MPa (*p* < 0.05) and so did the elongation at break, indicating a substantial drop from 53.6% to 41.6% (*p* < 0.05) with the addition of starch concentration from 2.5 wt.% to 7.5 wt.%. The significant decrement of 75.8% in tensile strength could be explained by the increase in amylopectin content as the starch concentration increased. The branched structure of amylopectin created a distance between the polymer chains resulting in weaker hydrogen bonds between them [21,34]. These research findings were in accord with the work of Sapei et al. [34] on the effects of different banana starch concentrations (10 to 30 vol.%) on the mechanical properties of chitosan-starch bioplastic film. It was worth noting that a very brittle bioplastic film was formed at 10 wt.% starch concentration. The brittleness incapacitated the tensile test, which can be evidenced from Table 1 exhibiting the cracked film with small broken pieces. This finding revealed that a 10 wt.% starch solution was too saturated to produce a smooth and uniform film whereas glycerol at 40 wt.% was insufficient to facilitate the formation of a film. The decrease in the elongation at break could be explained through the saturation phenomenon, whereby the bondings between the starch molecules increased as the starch concentration was increased to a saturation amount that would prevent glycerol molecules to fit into the molecular chains [35].

To gain an insightful overview of the starch synthesized bioplastic film and the effects on its mechanical properties, gelatinized duration required and appearance of film at diverse starch concentrations are presented in Table 1. In general, the surface of bioplastic film in contact with the container was shinny and glossy; meanwhile, the surface exposed to air during drying was dull looking. Starch is responsible for the cohesion of bioplastic during heating; a lower starch concentration required a longer heating process and would consume a greater energy consumption to evaporate the water content in achieving cohesion of bioplastic [36]. Despite that at 2.5 wt.% starch concentration, the bioplastic film possessed tensile strength that was 11.2% higher than that of 5 wt.% starch concentration, the gelatinization duration was 3.5 h longer. This implied 2.5 wt.% was not feasible to be considered as the optimum starch concentration from an economic perspective. Therefore, a 5 wt.% starch concentration was used to optimize the subsequent parameter investigated.

#### 3.1.2. Effect of Glycerol Loading

**Figure 3:**
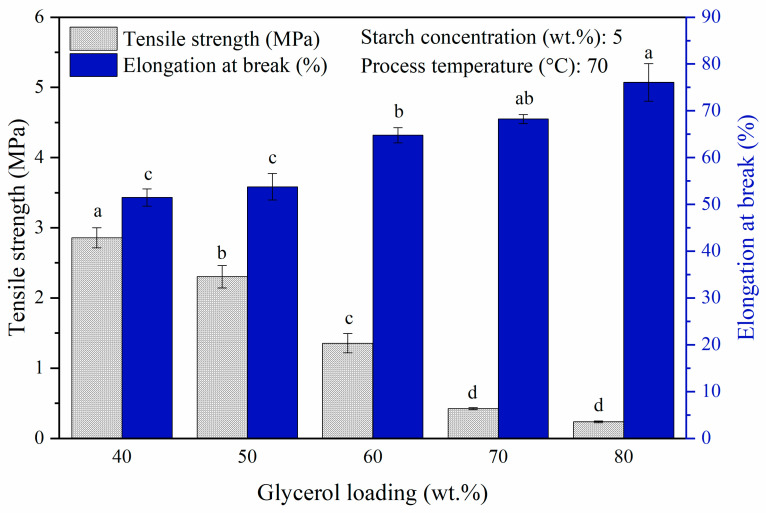
Tensile strength and elongation at break of pure starch-based bioplastic films with varying glycerol loadings. Different letters indicate the values are significantly different (*p* < 0.05).

Glycerol was used as a plasticizer to synthesize bioplastic in the present study to reduce the film brittleness by reducing the intermolecular forces between polymer chains, while increasing their mobility to produce a more elastic and flexible film [37]. From the findings of varying glycerol loadings (40 wt.% to 80 wt.% at 10 wt.% increments, which corresponded to starch: glycerol ratio (*w*/*w*) of 20:8 to 20:16) on the mechanical properties of the synthesized bioplastic film, the results in Figure 3 show that tensile strength exhibited a decreasing trend, in which it dropped from 2.86 MPa to 0.24 MPa when the glycerol loading was increased from 40 wt.% to 80 wt.%, and in contrast the elasticity of the film showed an opposite trend (*p* < 0.05). This could be reflected from the significant increase of 24.6% (from 51.5% to 76.1%) in the elongation at break value within the investigated range of glycerol loading. Higher glycerol loading provides greater free-movement space for the glycerol molecules to slip between the amylose-amylopectin chains. This weakens the interaction between the polymer in preventing the formation of rigid structures, while softening the polymer matrix, simultaneously [38]. The same behavioral plasticizing effect of glycerol on starch-based bioplastic film was observed by Ginting et al. [38] and Santana et al. [39] in the fabrication of durian seed starch-chitosan-sorbitol and cassava starch-glycerol films, respectively. Glycerol of 40 wt.% was used to optimize the subsequent parameters investigated, as the film obtained at lower amount of 30 wt.% glycerol was too brittle to be subjected for tensile test.

#### 3.1.3. Effect of Process Temperature

**Figure 4:**
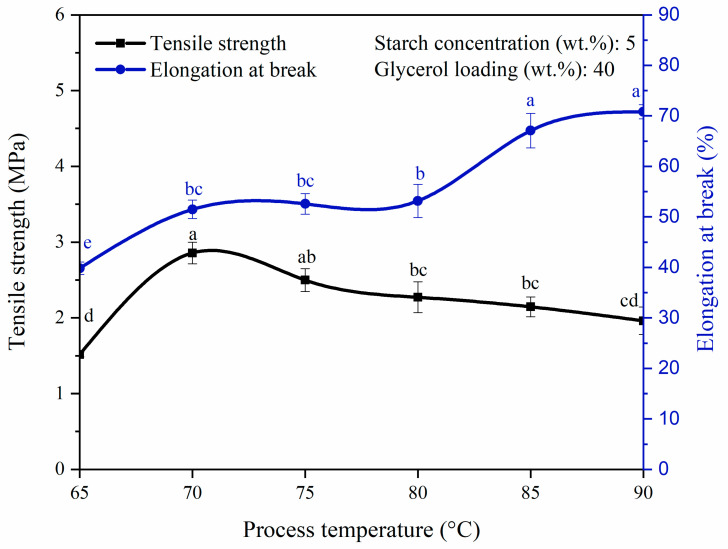
Tensile strength and elongation at break of pure starch-based bioplastic films at different process temperatures. Different letters indicate the values are significantly different (*p* < 0.05).

Temperature always exerts an effect on reaction. Preliminary study indicated that process temperature below 65 °C did not successfully produce a film. To elucidate the temperature effect on the fabrication of bioplastic, temperatures ranging from 65 °C to 90 °C were applied to the bioplastic solution and their effects on the mechanical properties of the bioplastic film are shown in Figure 4. When the process temperature increased from 65 °C to 70 °C, the tensile strength of the bioplastic film increased from 1.52 MPa to an optimum value of 2.86 MPa within the range of the investigated temperature (*p* < 0.05). The temperature increased from 65 °C to 70 °C had induced gelatinization by producing a film that is free of air bubbles, which can be observed from Table 1. However, tensile strength had then dropped to 1.96 MPa as the process temperature further increased to 90 °C (*p* < 0.05). The declining of tensile strength at higher process temperature might be caused by the weakening of intermolecular bonds in starch as excessive heating breaks the glycosidic bonds (bonds between monomers) in amylose [38]. Amylose plays an important role in the gel formation and the production of compact thin film. The excessive heat provided at 90 °C resulted in the depolymerization of long chain amylose into short chain amylose. This has subsequently decreased the amylose content [40,41]. A reduction in amylose content lowers the cohesiveness of bioplastics formation and, thus, its tensile strength is decreased [38].

Moreover, elongation at break increased with increasing process temperature. The highest investigation temperature of 90 °C gave rise to the longest elongation at break of 71.0%. At lower process temperature, the molecules in the polymer matrix are closely packed with lower kinetic energy. Thus, small free volume is present within the polymer matrix and the movement of the molecules are somewhat restricted. As the process temperature increases, the higher kinetic and thermal energies imparted to the polymer molecules could trigger more intense vibrations and generate more free volumes available for larger molecular chain rotation in the solution [42]. This suggests that besides the addition of glycerol, increase in the free volume between the polymer chains can also be achieved with increase in temperature. Eventually, the increasing mobility of starch molecules and the softening of film matrix would synergistically increase the flexibility of the synthesized film. This could be observed from the increase of 31.2% (39.8% to 71.0%) in the elongation at break percentage of bioplastic film as the process temperature was elevated from 65 °C to 90 °C (*p* < 0.05) [42,43]. Furthermore, Ginting et al. [38] also reported similar trends of tensile strength and elongation at break with respect to the process temperature. Both the optimum tensile strength and elongation at break, respectively, of 19 MPa and 2.6% were obtained at 70 °C when durian seed starch was used as matrix, sorbitol as plasticizer and chitosan as co-polymer.

#### 3.1.4. Effect of Chitosan Loading

**Figure 5:**
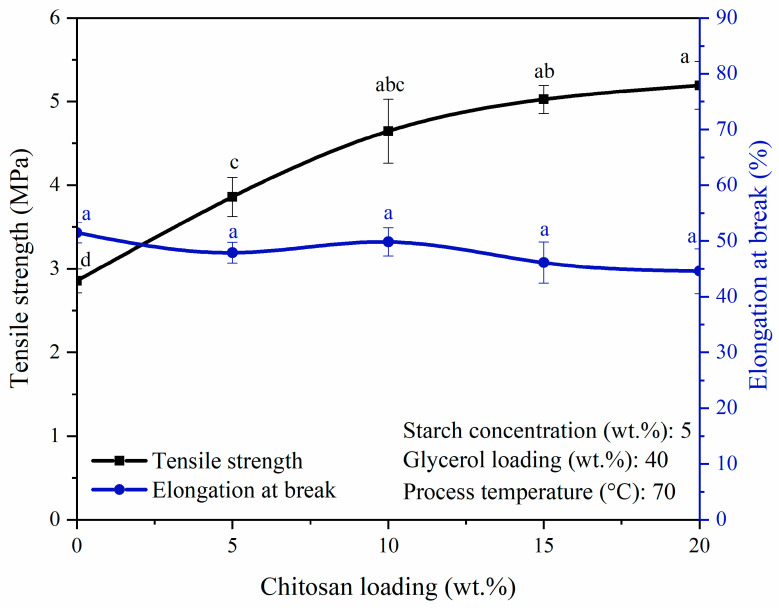
Tensile strength and elongation at break of bioplastic films at various chitosan loadings. Different letters indicate the values are significantly different (*p* < 0.05).

The effects of chitosan loading (0 wt.% to 20 wt.%, which corresponded to starch: glycerol: chitosan ratio (*w*/*w*) of 20:8:0 to 20:8:1) on the mechanical properties of bioplastic film are shown in Figure 5. With the addition of 5 wt.% chitosan loading, the tensile strength was improved to 3.86 MPa, which was 1.35 times greater than that of the pure starch-based bioplastic film at 2.86 MPa (*p* < 0.05). Further increase in the chitosan loading to 20 wt.% resulted in the tensile strength increasing to 5.19 MPa (*p* < 0.05). The reinforcement in the tensile strength could be attributed to the presence of more hydrogen bonds between NH_3_^+^ of the chitosan and OH^−^ of the corn starch. The amino groups (NH_2_) from chitosan were protonated to NH_3_^+^ in the acetic acid solution, while the orderly arranged crystalline structure of starch molecules was disrupted during the gelatinization process, resulting in the OH^−^ groups being exposed to form hydrogen bonds with NH_3_^+^ of the chitosan. Hence, chemical bonds of the bioplastic film were stronger and harder to be broken [27,38,44]. In contrast to the trend in tensile strength, elongation at break showed a decreasing trend with higher chitosan loading, although the perceived difference was not very significant (*p* > 0.05). Pure starch-based bioplastic film had elongation at break of 51.5%, whereas the chitosan-incorporated starch-based bioplastic film had elongation at break of 47.9% with 5 wt.% chitosan loading. When the chitosan loading was increased to 20 wt.%, the lowest elongation at break of 44.6% was obtained and similar results were reported by Hasan and Rahmayani [4] on the negative effect exerted by the increase in chitosan loading on the film flexibility. Another study published in the literature also demonstrated the improvement of tensile strength but lower elongation of break by 25 wt.% of chitosan loading in starch-based bioplastic film [27]. The tensile strength demonstrated in the published study [27] was higher than that of the present study (13.52 MPa vs. 5.19 MPa). In addition to the higher chitosan loading demonstrated in the published study (25 wt.% vs. 20 wt.%), this observation could be due to different plasticizers employed in both studies, in which sorbitol was employed. Sorbitol has six hydroxyl groups, which is double the number of hydroxyl groups compared to the glycerol. Sorbitol could bind more hydroxyl groups to the starch via hydrogen bonding, thereby achieving higher tensile strength than the present study [45].

In the present study, optimum tensile strength was determined at 20 wt.% chitosan loading. It is apparent that when the additional 5 wt.% chitosan loading was added, chitosan could not be dissolved completely in the starch solution leading to chitosan aggregation and the formation of numerous big lumps.

### 3.2. ANN Modeling Results

**Figure 6:**
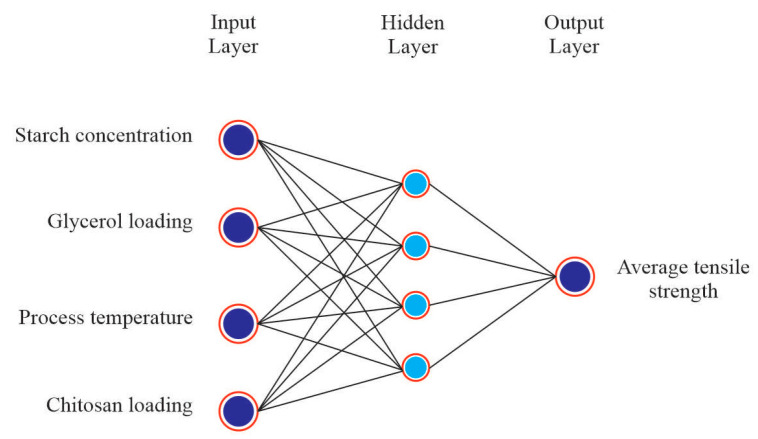
Architecture of ANN model for tensile strength.

**Figure 7:**
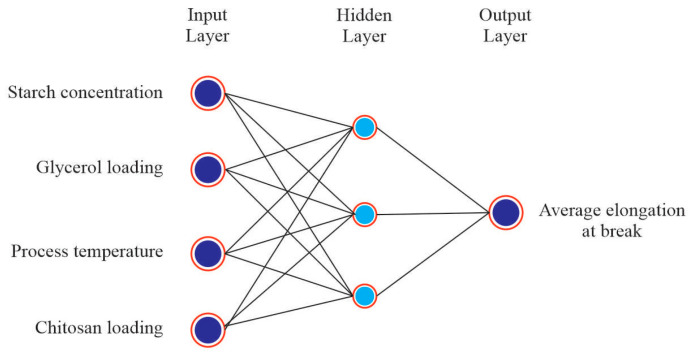
Architecture of ANN model for elongation at break.

**Figure 8:**
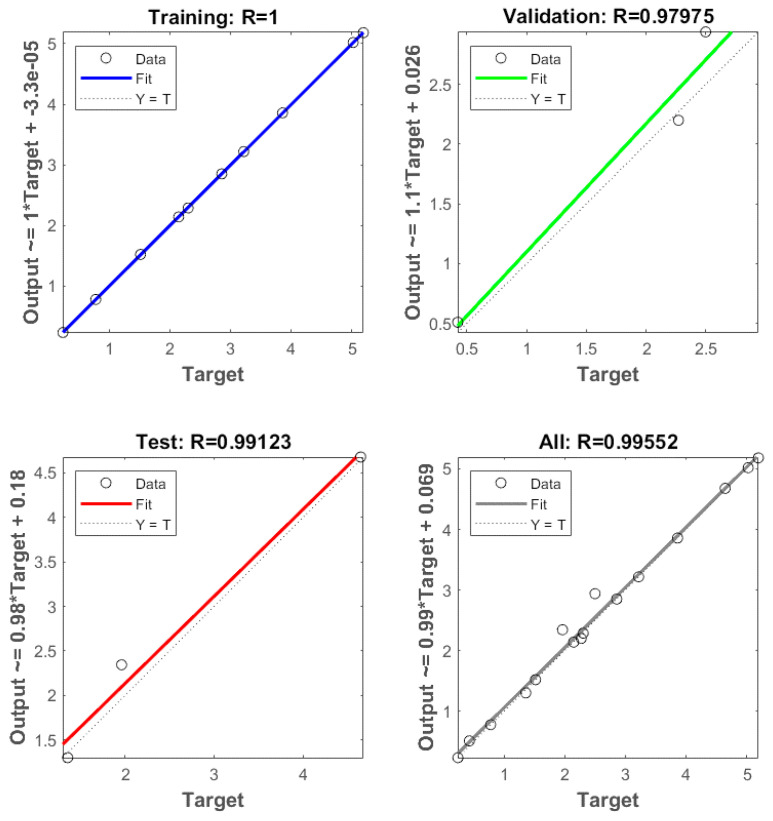
Coefficient of correlation (R) values of training, validation testing and overall datasets, for tensile strength.

**Figure 9:**
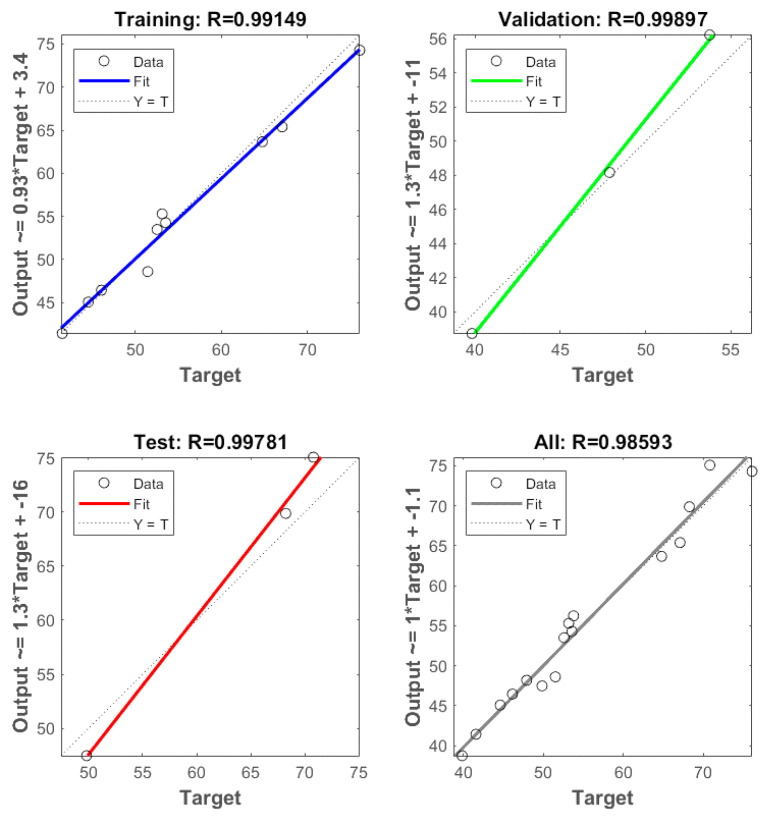
Coefficient of correlation (R) values of training, validation, testing and overall datasets, for elongation at break.

A total of 16 experimental runs were conducted using four input variables, i.e., starch concentration, glycerol loading, process temperature and chitosan loading and two output variables, i.e., average tensile strength and average elongation at break. The experimental data were randomly divided into 60% for the training, 20% for testing and 20% for validation. Based on the heuristic procedure, the optimum numbers of hidden neurons that fulfilled the requirements of minimum MSE and maximum R were 4 for tensile strength and 3 for elongation at break. Thus, the best topology was found to be 4-4-1 for tensile strength (Figure 6) and 4-3-1 for elongation at break (Figure 7) prediction. The values of R for training, validation, test and all, for tensile strength were 1, 0.97975, 0.99123 and 0.99552, respectively, (Figure 8). For elongation at break, the values of R for training, validation, test and all, were 0.99149, 0.99897, 0.99781 and 0.98593, respectively, (Figure 9). It could be observed from Table 2 and Table 3 that the values of R^2^ for tensile strength and elongation at break were 0.9955 and 0.9859, respectively, which were very close to unity. Therefore, it could be concluded that the model had good fit with the experimental data [46].

### 3.3. FTIR

**Figure 10:**
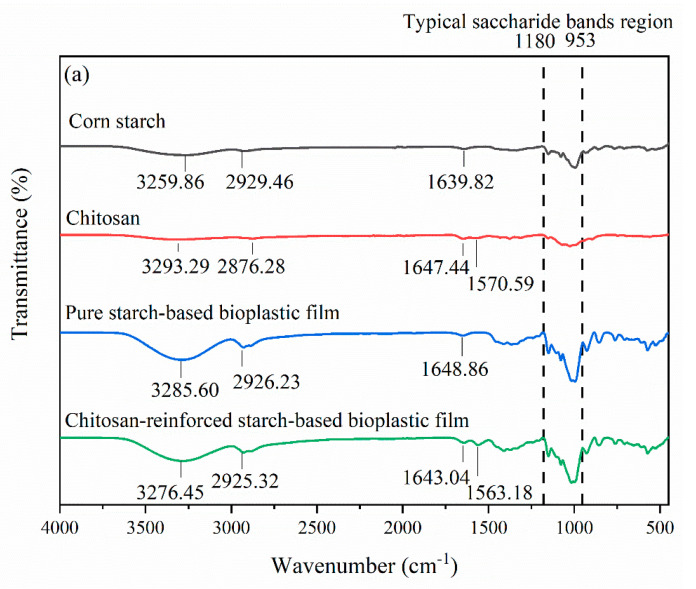

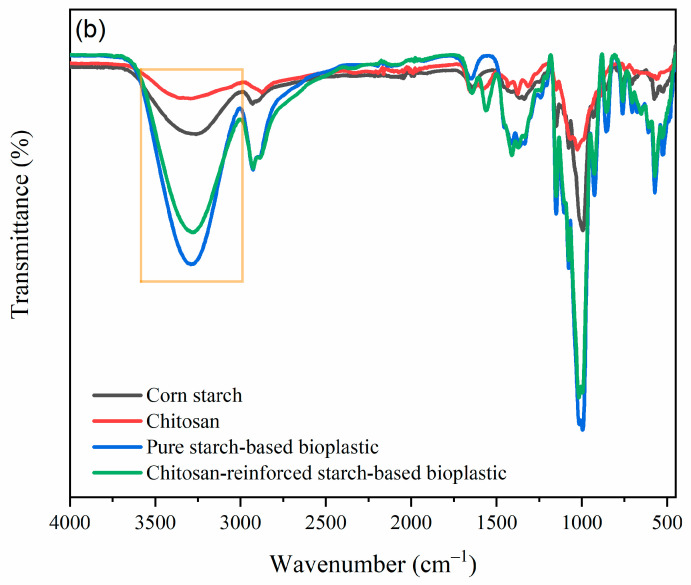
(**a**) FTIR spectra and (**b**) overlapping FTIR spectra of corn starch, chitosan powders, pure starch-based and chitosan-reinforced starch-based bioplastic films.

FTIR spectra of pure starch-based bioplastic film and chitosan-reinforced starch-based bioplastic films are compared with the spectra of corn starch and chitosan powders as shown in Figure 10a. The following bands observed in the corn starch powder at 3259.86 cm^−1^, 2929.46 cm^−1^ and 1639.82 cm^−1^ corresponded to O–H stretching, –CH_2_ stretching vibrations and O–H bending of water, respectively, [47]. Chitosan powder showed characteristic peaks at 3293.29 cm^−1^, which were attributed to N–H and O–H stretching, 1647.44 cm^−1^, which was assigned to amide I (C=O stretching) and 1570.59 cm^−1^, which was assigned to amide II (C–N stretching and C–N–H bending vibrations) [48]. In the spectra of pure starch-based bioplastic film, the peaks observed at 3285.60 cm^−1^ and 2926.23 cm^−1^ were associated with O–H and –CH_2_ stretching vibrations, respectively, [49]. The bands at 1648.46 cm^−1^ and 1411.63 cm^−1^ were, respectively, assigned to the O–H bending of water and CH_2_ groups [50]. The typical region of saccharide bands covered 1180–953 cm^−1^ in which the peaks at 1150.46 cm^−1^ and 1077.95 cm^−1^ were associated to stretching vibration of C–O in C–O–H groups, while the bands at 1015.68 cm^−1^ and 995.09 cm^−1^ were attributed to C–O in C–O–C groups [51,52].

Changes in FTIR spectrum were observed after chitosan was added to the starch-based bioplastic film. As compared with the chitosan powder, the amide I and amide II characteristic peaks of chitosan in the spectra of chitosan-reinforced starch-based bioplastic film were shifted from 1647.44 cm^−1^ to 1643.04 cm^−1^ and 1570.59 cm^−1^ to 1563.18 cm^−1^, respectively. The findings illustrated the interactions between hydroxyl groups of starch and amino groups of chitosan, which had promoted the compatibility of starch and chitosan [44,53]. Furthermore, the characteristic peak of inter- and intra-molecular hydrogen bonds in starch (3259.86 cm^−1^) and chitosan (3293.29 cm^−1^) were shifted to 3276.45 cm^−1^. This shift revealed the formation of inter- and intra-molecular hydrogen bonding between starch and chitosan [53]. Increase in the intensities of absorption peaks were noted in both pure starch-based and chitosan-reinforced starch-based bioplastic films relative to that of the corn starch and the chitosan powders as illustrated in the inset of Figure 10b. This was resulted from the overlapping of chemical bonds, indicating the presence of strong interaction between the molecules of different material components such as starch, chitosan and glycerol [54].

### 3.4. XRD

**Figure 11:**
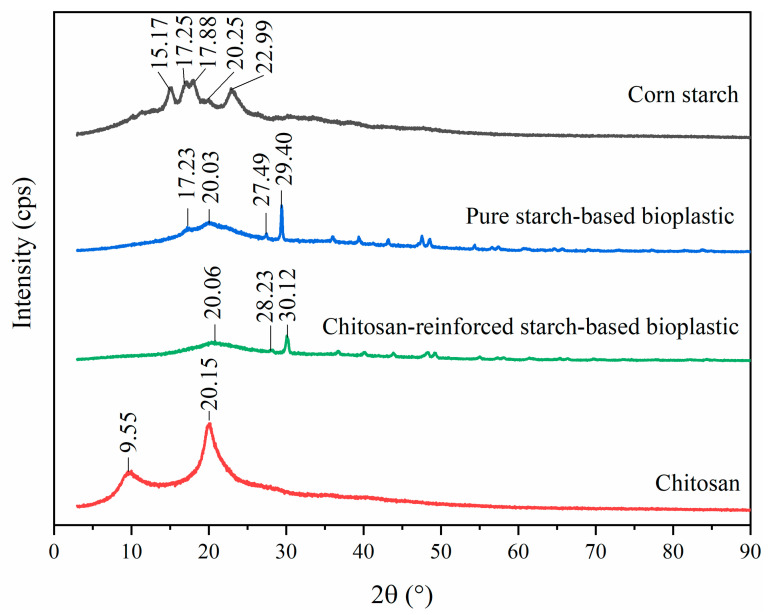
XRD profiles of corn starch, pure starch-based bioplastic film, chitosan-reinforced starch-based bioplastic film and chitosan.

Figure 11 presents the XRD profiles of corn starch powder, pure starch-based bioplastic film, chitosan-reinforced starch-based bioplastic film and chitosan powder. The corn starch powder had diffraction peaks at 15.17°, 17.25°, 17.88°, 20.25° and 22.99°, which corresponded to A-type crystalline structure [12,55,56]. Unlike that of the corn starch powder, the pure starch-based bioplastic film exhibited a different XRD pattern with peaks at 17.23°, 20.03°, 27.49° and 29.40°. This probably could be explained by the glycerol molecules that replaced the inter- and intramolecular hydrogen bonds, which had disrupted the crystallinity of starch during the bioplastic film fabrication [52]. The chitosan powder was in a crystalline state as two main diffraction peaks (2θ = 9.55° and 20.15°) were manifested in the XRD profile [44]. When chitosan was added into the pure starch-based bioplastic film, the crystallinity of the blend film (chitosan-reinforced starch-based bioplastic film) was decreased as reflected in the lower diffraction peaks at 20.06°, 28.23° and 30.12°. A similar finding was also discovered by other researchers [50,53,57]. The disappearance of peak at 17.23° in chitosan-reinforced starch-based bioplastic film could be considered as further evidence of the interaction between starch and chitosan [53]. In addition, the characteristic peak of chitosan (9.55°) did not appear in the chitosan reinforced starch-based bioplastic film. This could be due to the formation of intermolecular hydrogen bonding between starch and chitosan, which did not favor the crystallization of starch but had altered the chitosan structure [53]. These results further corroborated the FTIR results in which there was miscibility between corn starch and chitosan owing to their intermolecular interaction.

### 3.5. Thermal Analysis

**Figure 12:**
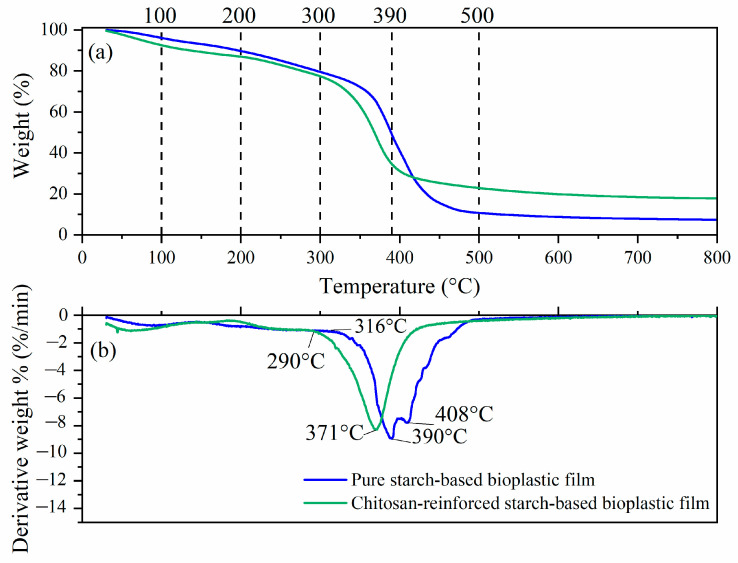
(**a**) TGA and (**b**) DTG curves of pure starch-based and chitosan-reinforced starch-based bioplastic films.

The TGA and derivative thermogravimetric (DTG) profiles of pure starch-based and chitosan-reinforced starch-based bioplastic films are shown in Figure 12a,b, respectively. There are five weight losses in both films. The first weight loss occurred between ~30 °C and ~100 °C was associated with the evaporation of free water [18]. The second weight loss took place between ~100 °C and ~200 °C was related to the evaporation of moisture from the bioplastic films [58]. The third weight loss range of ~200 °C to ~300 °C corresponded to the thermal decomposition of starch in bioplastic films and degradation of glycerol (boiling point of 290 °C) [18]. Starch contained amylose particles, which could form carbon, hydrogen and oxygen in its volatile state [59]. Around 300 °C to ~500 °C, compounds with lower molecular weight such as plasticizer (glycerol) and additive (chitosan) were released as well as the degradation of starch which took place at 408 °C, as shown in Figure 12b. Beyond 500 °C, pyrolysis of carbonated compounds took place resulted in inorganic materials in the remaining residue samples [60].

From the DTG curve as shown in Figure 12b, the decomposition temperatures of pure starch-based and chitosan reinforced starch-based bioplastic films were recorded at 390 °C and 371 °C, respectively. The latter film had a much lower decomposition temperature, which might be attributed to the presence of acetic acid that disrupted the inter- and intra-molecular hydrogen bonds to induce thermal degradation [61,62]. Another reason could be ascribed to the decreased crystallinity in the chitosan-reinforced starch-based bioplastic film, as previously discussed in the XRD results. Film with lower crystallinity would have more molecular movement in the polymer chains and thereby lower the degradation temperature [63,64]. As approaching 900 °C, the chitosan-reinforced starch-based bioplastic film was found to have 17% residue, which was 10% higher than pure starch-based bioplastic film with 7% residue. From the DTG curves, as shown in Figure 12b, the results suggested that pure starch-based and chitosan-reinforced starch-based bioplastic films should be subjected to applications below 316 °C and 290 °C, respectively, without degradation or loss of their characteristics. Most of the applications associated with this type of bioplastic, such as packaging and containers, are usually operated from room temperature to slightly higher than 100 °C. These operating temperatures are well below the thermal stability of the bioplastic films.

### 3.6. Water Uptake Test

**Figure 13:**
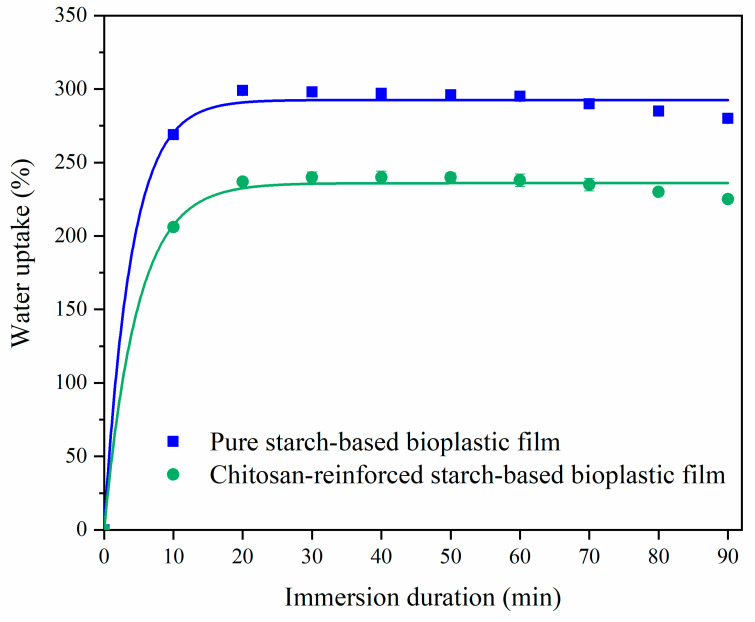
Water uptake percentages of pure starch-based and chitosan-reinforced starch-based bioplastic films at various immersion durations.

Water uptake capability of bioplastic film is directly related to its hydrophilic nature [37]. The water uptake trends of pure starch-based and chitosan-reinforced starch-based bioplastic films of the study are depicted in Figure 13. Comparatively, chitosan-reinforced starch-based bioplastic film had a lower water uptake capability. Both types of bioplastic films absorbed water rapidly within the first 10 min of water immersion, indicating their strong hydrophilic nature. This was due to the abundance of free hydroxyl groups available in starch and chitosan, which would allow large amount of water to penetrate into the film within minutes [10]. After 10 min of immersion time, starch-based bioplastic film absorbed 270% water and chitosan-reinforced starch-based bioplastic film absorbed 206% water. Beyond 40 min immersion period, pure starch-based bioplastic film reached its maximum water uptake of 302%, whereas the chitosan-reinforced starch-based bioplastic film only achieved a maximum water uptake of 251% after 50 min immersion. Once exceeded their respective optimum uptake threshold, both the pure starch-based and chitosan-reinforced starch-based bioplastic films showed a gradual decrease in water uptake and mass losses. The pure starch bioplastic film can be dissolved in water faster than chitosan-reinforced starch-based bioplastic film, indicating that the latter film possesses better stability in water. When the water immersion time was prolonged to 90 min, both pure starch and chitosan-reinforced starch-based bioplastic films showed a decrease in water uptakes at 281% and 217%, respectively. Comparing the maximum water uptake capability between pure starch-based bioplastic film (302%) and chitosan-reinforced starch-based bioplastic film (251%), the larger water uptake capability demonstrated by the latter suggesting that chitosan could be used to improve the water resistance of pure starch-based bioplastic film attributing to the hydrophilic nature of amylose. Water molecules were easily absorbed by the pure starch-based bioplastic film during immersion [4]. With the incorporation of chitosan into the starch-based bioplastic film, the interlink network formed between starch and chitosan could prevent water molecules penetrating through the film [10]. Additionally, from another perspective, water resistance of bioplastic film was enhanced by the hydrophobic nature of chitosan, which has mitigated the hydrophilic nature of starch, diminishing their interactions with water molecules [4,65]. The observation is in agreement with the work of Anggraini et al. [21] on sago starch-chitosan-sorbitol bioplastic film reporting a water uptake of 204.97% for bioplastic film without chitosan, and a much reduced water uptake of 130.31% in 20 wt.% chitosan-loaded bioplastic film.

### 3.7. Biodegradation Test

**Figure 14:**
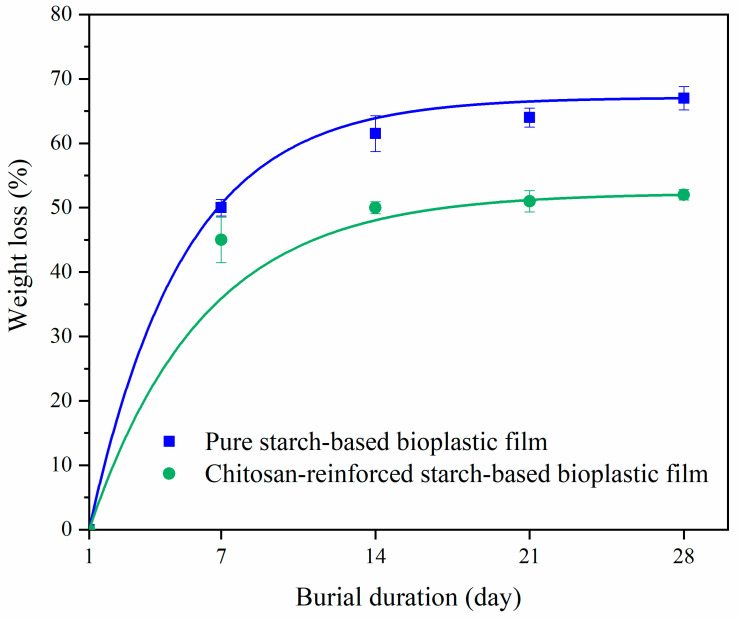
Weight loss percentages of pure starch and chitosan-reinforced starch-based bioplastic films during burial period.

The percentage weight losses of pure starch-based and chitosan-reinforced starch-based bioplastic films are displayed in Figure 14. The weight losses of both bioplastic films increased with the burial time, suggesting the degradation of bioplastic films. On day 7, both films were found to have degraded by more than 40% of their initial weight. Pure starch-based bioplastic film degraded by 53.6% and chitosan-reinforced starch-based bioplastic film degraded by 47.5%. There was no significant weight loss for both bioplastic films on the 2nd and the 3rd week. On day 28, the weight losses of pure starch-based and chitosan-reinforced starch-based bioplastic films, respectively, reached 67.7% and 52.1%. Pure starch-based bioplastic film showed more weight loss than the chitosan-reinforced starch-based bioplastic film throughout the burial period, which could be due to higher hydrophilicity of the starch matrix [66]. The chitosan-reinforced starch-based bioplastic film had slower degradation, which might be due to the antimicrobial properties of chitosan as well as the interaction between starch and chitosan via hydrogen bonding, which possibly reduced the hydrophilicity of the starch matrix and thus slowing down the biodegradation rate [34,67].

## 4. Conclusions

Chitosan-reinforced starch-based bioplastic film was successfully prepared through the solution casting and evaporation method. This study described the effects of various parameters in attaining the optimum tensile strength of bioplastic film, and also confirmed the relationship between starch concentration and process temperature with the gelatinized duration. Higher starch concentration and process temperature would shorten the gelatinized duration and negatively affect the tensile strength. Using the hydrophobic nature of chitosan as a reinforcement agent not only could help to enhance the tensile strength, but also could improve the water resistance of starch-based bioplastic film. Although chitosan-reinforced starch-based bioplastic film could only be subjected to application below 290 °C, which was lower than pure starch-based bioplastic film (316 °C), it exhibited more than 50% faster biodegradability property, which will be greener for the environment.

This study has also laid the foundation in developing similar types of biodegradable chitosan-reinforced starch-based bioplastic film, which could be used to replace petroleum-based plastics. The formulation of composition based on corn starch as a matrix would also be beneficial in designing a more complete experimental study using starch derived from other agricultural waste. Although the present study demonstrated that the mechanical properties of bioplastic film were affected by the starch concentration, the relationship between the starch concentration and the mechanical properties of the bioplastic films is still not conclusive. Therefore, more empirical studies are required to refine and improve the reported findings.

## Figures and Tables

**Table 1:** Gelatinized duration required and film appearance at different starch concentrations.

Starch Concentration (wt.%)	Gelatinized Duration (h)	Film Appearance
2.5	5.5	Film free of air bubbles. 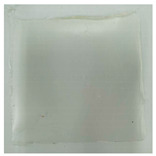
5	2	Film free of air bubbles. 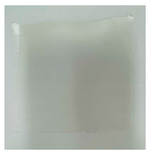
7.5	1.5	Film with tiny air bubbles. 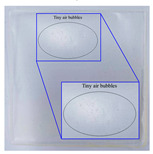
10	1	Brittle film. 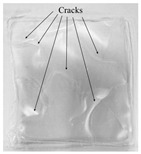

**Table 2:** Comparison of the experimental tensile strength and predicted tensile strength by ANN model.

Experimental Run	Starch Concentration (wt.%)	Glycerol Loading (wt.%)	Process Temperature (°C)	Chitosan Loading (wt.%)	Experimental Tensile Strength (MPa)	Predicted Tensile Strength (MPa)
1	2.5	40	70	0	3.22	3.22
2	5	40	70	0	2.86	2.85
3	7.5	40	70	0	0.78	0.78
4	5	50	70	0	2.30	2.29
5	5	60	70	0	1.36	1.30
6	5	70	70	0	0.42	0.51
7	5	80	70	0	0.24	0.23
8	5	40	65	0	1.52	1.52
9	5	40	75	0	2.50	2.94
10	5	40	80	0	2.27	2.20
11	5	40	85	0	2.15	2.14
12	5	40	90	0	1.96	2.34
13	5	40	70	5	3.86	3.86
14	5	40	70	10	4.65	4.68
15	5	40	70	15	5.03	5.02
16	5	40	70	20	5.19	5.18
*R* ^2^	0.9955					

**Table 3:** Comparison of the experimental elongation at break and predicted elongation at break by ANN model.

Experimental Run	Starch Concentration (wt.%)	Glycerol Loading (wt.%)	Process Temperature (°C)	Chitosan Loading (wt.%)	Experimental Elongation at Break (%)	Predicted Elongation at Break (%)
1	2.5	40	70	0	53.6	54.3
2	5	40	70	0	51.5	48.6
3	7.5	40	70	0	41.6	41.4
4	5	50	70	0	53.8	56.2
5	5	60	70	0	64.8	63.7
6	5	70	70	0	68.2	69.9
7	5	80	70	0	76.1	74.3
8	5	40	65	0	39.8	38.7
9	5	40	75	0	52.6	53.5
10	5	40	80	0	53.2	55.3
11	5	40	85	0	67.1	65.4
12	5	40	90	0	70.8	75.1
13	5	40	70	5	47.9	48.2
14	5	40	70	10	49.8	47.5
15	5	40	70	15	46.1	46.4
16	5	40	70	20	44.6	45.1
*R* ^2^	0.9859					

## Data Availability

The data presented in this study are available on request from the corresponding author.
